# Effects of Intercooling Intensity on Temperature Field and Microstructure of Large-Scale 2219 Al Alloy Billet Prepared by Internal Electromagnetic Stirring Casting

**DOI:** 10.3390/ma15051809

**Published:** 2022-02-28

**Authors:** Yang Qiu, Xintao Li, Mingyang Liu, Nan Zhou, Kaihong Zheng

**Affiliations:** 1 Institute of New Materials, Guangdong Academy of Sciences, Guangzhou 510630, China; lxt_hq@163.com (X.L.); liumingyang4671@163.com (M.L.); zhounan@gdinm.com (N.Z.); zhkaihong2003@163.com (K.Z.); 2 Guangdong Provincial Key Laboratory of Metal Toughening Technology and Application, Guangzhou 510630, China

**Keywords:** large-scale billet, internal electromagnetic stirring, intercooling intensity, temperature field, microstructure

## Abstract

Internal electromagnetic stirring is an advanced melt treatment method, which can be used in direct chill casting to prepare large-scale Al alloy billets. Intercooling intensity is a primary parameter of internal electromagnetic stirring; its effects on temperature fields and microstructures have been investigated via numerical simulations and industrial experiments, respectively. The simulated results show an increase in the intercooling affected area and a decrease in sump depth with an increase in the intercooling heat transfer coefficient. The heat transfer coefficient should not exceed 500 W/(m^2^ °C) because the solid fraction of the intercooling end bottom may exceed 50%. The experiment’s results demonstrate that the average grain sizes in the edge, 1/2 radius, and center are 151 ± 13 μm, 159 ± 14 μm, and 149 ± 16 μm, respectively, under a liquid nitrogen flow rate of 160 L/min, which is much finer than that of 80 L/min and more homogeneous than that of 240 L/min. Furthermore, an experimental liquid nitrogen flow rate of 80 L/min, 160 L/min, and 240 L/min approximately correspond to the simulated heat transfer coefficient of 200 W/(m^2^ °C), 300 W/(m^2^ °C), and 400 W/(m^2^ °C), respectively.

## 1. Introduction

Coarse and inhomogeneous microstructures always occur during conventional direct chill casting (DCC) of billets, an issue that usually worsens as the scale of the billet and range of solidification increases [1,2,3,4]. Deformation processes, such as rolling and forging, can refine a microstructure, but cannot significantly improve its inhomogeneity [5]. On the contrary, under certain conditions deformation may actually aggravate the microstructure’s inhomogeneity. When the strain temperature is low and the strain rate is high, some defects, such as cracking, flow localization, and an adiabatic shear band may occur, resulting in poor workability of as-cast materials and lower relative mechanical properties in as-deformed materials [6,7,8]. Hence, it is vital to obtain fine and homogeneous microstructures in billets [9,10,11].

A large number of studies have shown that the abovementioned problems can be solved by using electromagnetic stirring (EMS) during DCC [12,13,14,15]. However, the skin effect applies to conventional EMS, demonstrating that the penetration depth of the magnetic field into the melt is limited [16]. The magnetic induction intensity in the center melt is too small to generate effective electromagnetic stirring effects. Therefore, conventional EMS can only be applied to small-scale billets. To solve this problem, the annular EMS method was proposed; inserting a core into the melt center [17,18]. An annular gap is formed between the core and the hot top mold, avoiding the weak stirring area and greatly increasing the shear strength of the electromagnetic force. However, annular EMS does not fundamentally change the traditional heat transfer pattern (from outside to inside); the melt temperature field is always very inhomogeneous, especially in large-scale billets. Therefore, a uniform DCC method, which involves inserting an in-mold cooler (with a cooling function) into the melt center [19,20], is proposed. The cooler occupies the melt center, which reduces the temperature of the center melt to a certain extent while, simultaneously, most of the heat at the liquid sump can be continuously removed by passing a cooling medium into the cooler. In addition, the existence of an annular also accelerates the forced convection generated by the EMS. Based on uniform DCC, the internal EMS (IEMS) method, which integrates EMS and intercooling, was proposed as a more advanced and convenient method [21,22]. Compared to uniform DCC, the stirring pattern is changed from the outside-to-inside of conventional EMS to the inside-to-outside of IEMS. Large-scale 2219, 7050, and 7075 Al alloy billets with fine and inhomogeneous microstructures have been obtained via IEMS DCC (internal electromagnetic stirring direct chill casting) [21,22,23].

As an Al-Cu-Mn series alloy, the 2219 aluminum alloy has high weldability, fracture toughness, and stress corrosion resistance [24,25]. It also maintains excellent strength and toughness from temperatures as low as −250 °C to those as high as 300 °C. Hence, it is widely used in the aerospace industry, especially in the storage tanks of space vehicles [26,27]. In this paper, a 2219 Al alloy billet, with a diameter of 508 mm, was chosen as the researched material. As intercooling intensity is one of the most important parameters for the IEMS DCC process, numerical simulations and industrial experimentations, subject to different intercooling intensities, will be carried out in order to investigate its effects on temperature fields and microstructures.

## 2. Materials and Methods

**Figure 1:**
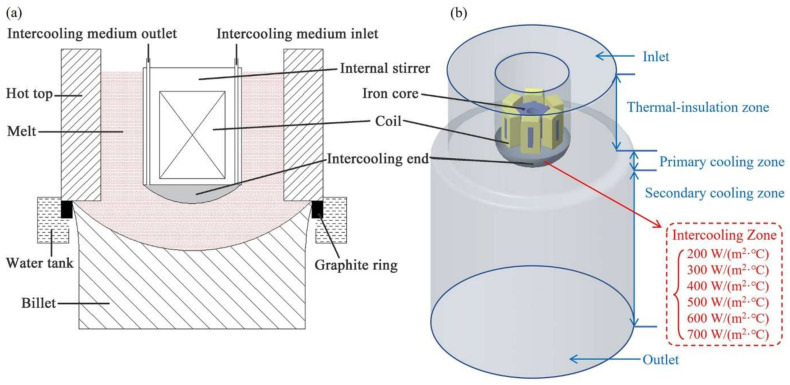
IMES DCC process: (**a**) two-dimensional schematic; (**b**) three-dimensional geometry models and boundary conditions.

Figure 1 shows a two-dimensional schematic and a three-dimensional geometric model of the IMES DCC process. To simplify the mathematical model and decrease calculations, the following assumptions were made: (1) the displacement current was ignored; (2) the Joule heat was ignored; (3) the effects of melt velocity changes on electromagnetic field distribution was ignored; (4) the melt is an incompressible fluid; (5) all of the melt is in its homogeneous phase [28,29].

### 2.1. Governing Equations

The simulation is coupled with the temperature field (including solidification), flow field, and electromagnetic field. Hence, the following governing equation should be solved:

Continuity equation:

(1) $$\frac{\partial \left(\rho u_{i}\right)}{\partial x_{i}}=0$$

Momentum equation:

(2) $$\rho \frac{\partial u_{i}u_{j}}{\partial x_{j}}=\frac{\partial }{\partial x_{j}}\left[\mu_{eff}\left(\frac{\partial u_{i}}{\partial x_{j}}+\frac{\partial u_{j}}{\partial x_{i}}\right)\right]-\frac{\partial P}{\partial x_{i}}+\rho g+F+S$$

in which the ρ, $\mu_{eff}$, P, F, g, and S are density, the effective viscosity coefficient, pressure, Lorentz force, gravity acceleration, and source term generated by dendrite flowing in the mushy zone, respectively. The $u_{i}$ and $u_{j}$ are velocity components in the $x_{i}$ and $x_{j}$ direction, respectively. The $x_{i}$ and $x_{j}$ are coordinates in the $x_{i}$ and $x_{j}$ direction, respectively.

(3) $$S=\frac{{\left(1-f_{L}\right)}^{2}}{\left(f_{L}^{3}+\delta \right)}A_{mush}\left(u-u_{s}\right)$$

(4) $$f_{L}=\left\{\begin{array}{c} 0\;\left(T\leq T_{S}\right)\;\\ 1-\frac{1}{1-k_{P}}\frac{T_{L}-T}{T_{f}-T}\;(T_{S}<T<T_{L})\\ 1\;\left(T\geq T_{L}\right) \end{array}\right.$$

in which the δ is minima (ensuring the denominator is not equal to 0); $A_{mush}$, $u_{s}$, and $f_{L}$ are mushy zone constant, casting velocity, and liquid fraction, respectively, and $k_{P}$, $T_{S}$, $T_{L}$, and $T_{f}$ are solute partition coefficient, solidus, liquidus, and pure metal melting temperature, respectively.

Energy equation:

(5) $$\frac{\partial }{\partial t}\left(\rho h\right)+\nabla \cdot \left(\rho hu\right)=\nabla \cdot \left(k_{t}\nabla T\right)+S_{h}$$

(6) $$h=\int_{298.15K}^{T}c_{p}dT$$

in which the h, $k_{t}$, $S_{h}$, and $c_{p}$ are enthalpy, thermal conductivity, solidification latent heat, and specific heat, respectively.

The electromagnetic field governing equations include Maxwell’s equations and Ohm’s Law:

(7) ∇×H=J

(8) $$\nabla \times E=-\frac{\partial B}{\partial t}$$

(9) ∇⋅B=0

(10) J=σE

in which the H, J, E, B, and σ are magnetic field intensity, induced current density, electric field intensity, magnetic induction intensity, and electrical conductivity, respectively.

The Lorentz force can be calculated by the following relationship:

(11) F=J×B

The magnetic field was calculated by Maxwell software (v16, 2012, Ansoft Co., Pittsburgh, PA, USA) and then imported to the Magnetohydrodynamics (MHD) module of ANSYS Workbench Fluent. After having been calculated, the temperature field, including temperature distribution, sump morphology, and depth would be presented in the CFD-Post module of the Workbench.

### 2.2. Boundary Conditions

The boundary conditions were set up approximately, according to the IEMS DCC process. As shown in Figure 1b, the boundary conditions of simulated areas are defined in blue, while the intercooling area is defined in red. The inlet and outlet were set as velocity inlet and velocity outlet. In the hot top area, a thermal-insulation wall was set up. In order to more accurately simulate the process of heat transference, user-defined functions were compiled and applied to the primary and secondary cooling zones.

In the primary cooling zone, solidification shrinkage occurred when the melt made contact with the graphite ring. An air gap occurred, causing a decrease in the heat transfer coefficient. According to the conclusions of Caron’s research, and considering the solidus and liquidus of the 2219 Al alloy, the heat transfer coefficient in the primary cooling zone can be expressed by [30]:

(12) $$h=\left\{\begin{array}{c} 1500\;\;\;\;,\;\;\;\;\;\;\;\;\;\;\;\;\;(T>916\;K)\\ 28.5T-24,600\;\;,\;\;\;\;\;\;\;\;\;\;\;\;\left(916\;K\geq T\geq 866\;K\right)\\ 75\;\;\;\;\;\;\;\;,\;\;\;\;\;\;\;\;\;\;\;\;\;(T<866\;K) \end{array}\right.$$

In the secondary cooling zone, the heat transfer is very complicated. Forced convection, nucleate boiling, transition boiling, and film boiling may occur, depending on the temperature of the billet’s surface. According to the results of Weckman’s research, the heat transfer coefficient can be simplified down to the following two conditions [31]:

when nucleate boiling occurs,

(13) $$h_{b}=\left(-167,000+704\bar{T}\right)Q^{1/3}+\frac{20.8\Delta T_{x}^{3}}{\Delta T}$$

when forced convection occurs,

(14) $$h_{c}=\left(-167,000+704\bar{T}\right)Q^{1/3}$$

A criterion can be used to judge whether nucleate boiling occurs:

(15) $$h_{i}=3910{\left(\Delta T_{x}\right)}^{2.16}$$

in which the $\bar{T}$ is the average temperature of cooling water, Q is the flow rate of cooling water in the unit’s perimeter, $\Delta T_{x}$ is the difference between billet surface temperature and water saturation temperature, and ΔT is the difference between billet surface temperature and cooling water temperature.

The heat transfer coefficient in the secondary cooling zone can be expressed by:

(16) $$h=\left\{\begin{array}{c} h_{c},\;\;\;|\;\;\;h_{i}\leq h_{c}\\ h_{b},\;\;\;|\;\;\;h_{i}>h_{c} \end{array}\right.$$

In the intercooling zone, intercooling intensity was characterized by the heat transfer coefficient and then acted on the bottom contact area of the intercooler and melt (intercooling end). The intercooling medium and the inner surface of intercooling end contact. Then the heat transfer occurs. The abovementioned heat flux can be approximately equivalent to the melt heat transfer via the intercooling end. Hence, the heat transfer coefficient can be calculated by [32]:

(17) $$h=\frac{mc\left(t_{1}-t_{2}\right)F_{1}}{\left(t_{3}-t_{4}\right)F_{2}}$$

in which m, c, $t_{1}$, $t_{2}$, and $F_{1}$ are mass flux pre-unit area, specific heat capacity, outflow temperature, inflow temperature, and flow channel cross-section area of intercooling medium, respectively, and $t_{3}$, $t_{4}$, and $F_{2}$ are outer surface temperature, inner surface temperature, and surface area of intercooling end, respectively.

### 2.3. Materials and Properties

**Figure 2:**
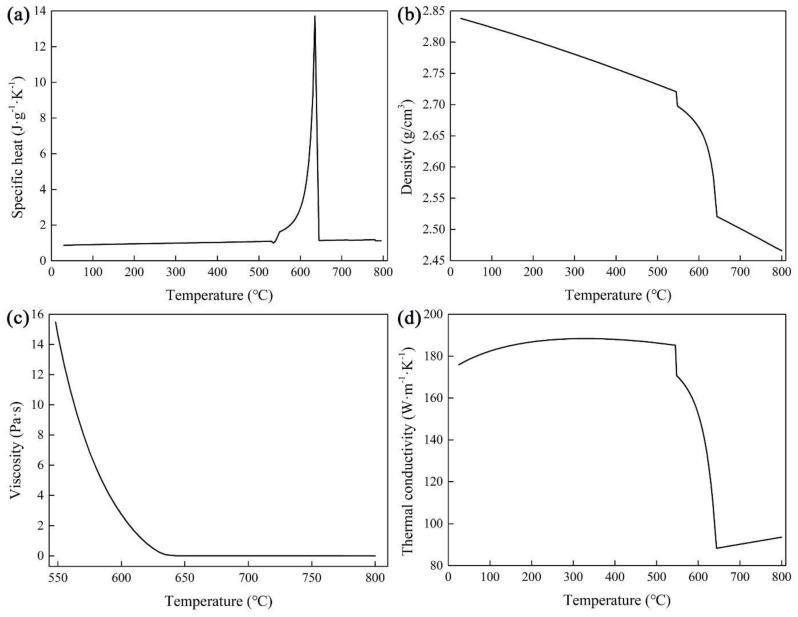
Thermal properties of Al−6.35Cu−0.32Mn−0.13V−0.18Zr−0.06Ti alloy. (**a**) Specific heat; (**b**) Density; (**c**) Viscosity; (**d**) Thermal conductivity.

The 2219 Al alloy to be used for simulation and experimentation is Al-6.35Cu-0.32Mn-0.13V-0.18Zr-0.06Ti. 15. Points of different positions were measured to determine its average chemical composition using a direct reading spectrometer (Oxford Instrument Foundry-Master Pro, Oxford, Great Britain). Thermal properties were calculated by JMatPro software (7.0, 2017, Sente Software Ltd., Guildford, Great Britain) and are shown in Figure 2. Meanwhile, its solidification, latent heat, electrical conductivity, and relative magnetic permeability are 385,000 J/kg, 3,800,000 S/m, and 1, respectively. For the electromagnetic stirrer, the relative magnetic permeability of copper coils and iron cores are 1 and 2000, respectively.

### 2.4. Experimentation

The pure industrial Al, pure industrial Cu, Al-10Mn, Al-10V, and Al-10Zr master alloys were melted in a medium frequency induction furnace and then transferred to a holding furnace. After compositional adjustments, melt purification, and refinement (add Al-5Ti-B), we began stirring in the bottom of the furnace to promote the homogeneous distribution of compositions. When the grain refiner had been completely melted, and the melt temperature had decreased to the casting temperature, DCC began. The melt temperature of the hot top was 650 °C, the casting speed was 0.0004 m/s, and the second water-cooling rate was 120 L/min. For the IEMS DCC, the internal stirrer was inserted into the hot top center, in which the intercooling end bottom was 10 mm below the graphite ring top. The alternating current ampere-turns and frequency were 1000 At and 20 Hz, respectively. It should be noted that the side surface of the internal stirrer was covered by thermal insulation material. Hence, no heat transfer occurs in this zone. All of the parameters applied to the simulation are the same as the abovementioned. The differences between the experiment’s parameters and those of the simulation are brought about due to the type of intercooling intensity used; the former is a flow rate of intercooling liquid nitrogen (80–240 L/min), while the latter is heat transfer coefficient (200–700 W/(m °C)).

The magnetic induction intensity in the half-height of the coil was tested using a gauss meter. The actual results were compared with the simulated results. Meanwhile, the microstructures undergoing different intercooling intensities were anode coated in a 3% HBF_4_ aqueous solution under direct voltage, 30 V for 20 s, and then observed using a Zeiss Axiovert 200 MAT optical microscope (Carl Zeiss Microscopy GmbH, Jena, Germany). The average grain size was determined using the linear intercept method (ASTM E112-10). The second phases were observed using a ZEISS GeminiSEM 300 (Carl Zeiss Microscopy GmbH, Jena, Germany). The mechanical properties were tested using a SHIMADZU AG-250kN IS tensile machine (SHIMADZU Co., Kyoto, Japan).

## 3. Results

### 3.1. Magnetic Induction Intensity

**Figure 3:**
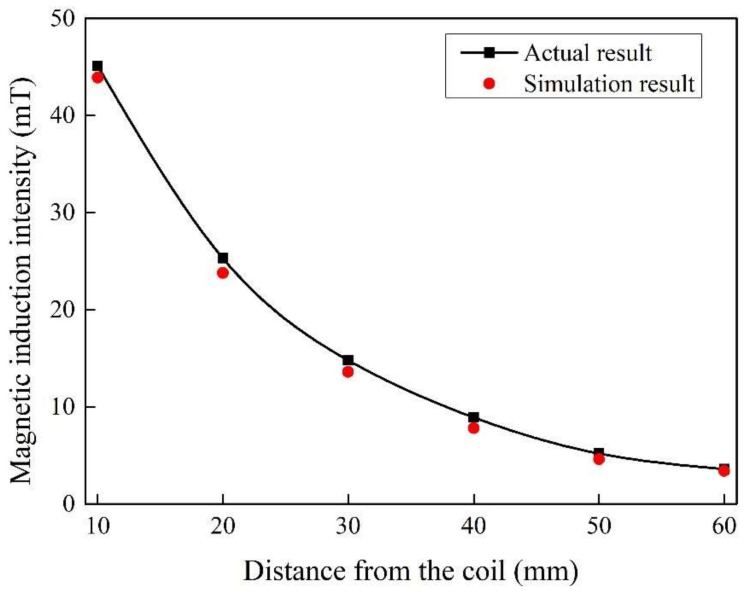
Comparison of the actual and simulated results for magnetic induction intensity.

Figure 3 shows the magnetic induction intensity of different distances from the coil under alternating current ampere-turns 2500 At and frequency 20 Hz, respectively. The actual results and simulated results appear to verify similar trends as the distance from the coil increases. Moreover, the differences between the two are relatively small, which demonstrates the accuracy and credibility of the simulation.

### 3.2. Temperature Field

**Figure 4:**
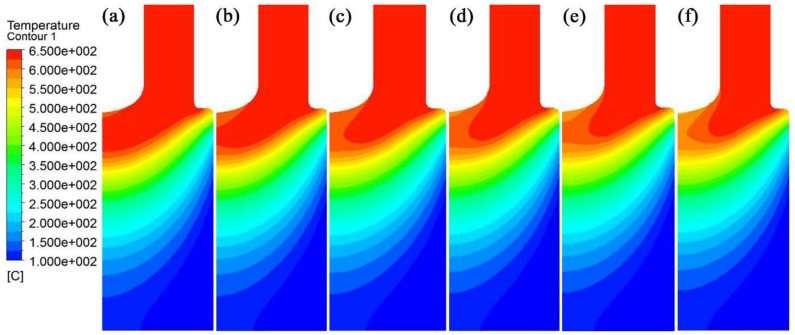
Temperature distribution under different intercooling heat transfer coefficients: (**a**) 200 W/(m^2^ °C); (**b**) 300 W/(m^2^ °C); (**c**) 400 W/(m^2^ °C); (**d**) 500 W/(m^2^ °C); (**e**) 600 W/(m^2^ °C); (**f**) 700 W/(m^2^ °C).

Temperature distribution in the longitudinal section of the IEMS DCC billet is shown in Figure 4. When the intercooling heat transfer coefficient is 200 W/(m^2^ °C), the intercooling effect is weak, and the temperature decreased area is only in the nearby intercooling end. As the intercooling intensity increases, its affected area of increases, and the temperature of the center melt decreases gradually. When the heat transfer coefficient reaches 700 W/(m^2^ °C), the affected distance of intercooling increases significantly in a vertical direction, and the affected radius in the horizontal direction also increases accordingly.

**Figure 5:**
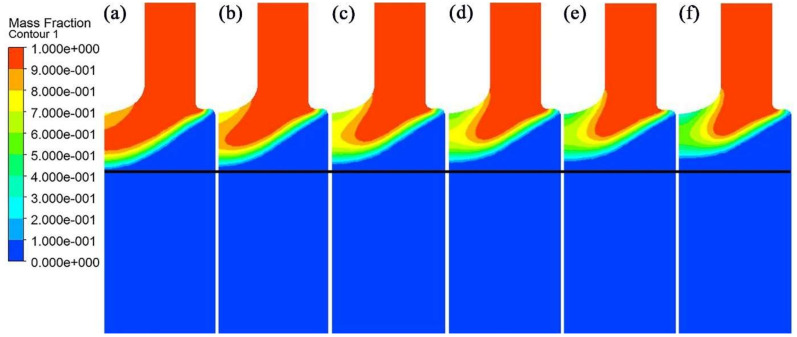
Sump under different intercooling heat transfer coefficients: (**a**) 200 W/(m^2^ °C); (**b**) 300 W/(m^2^ °C); (**c**) 400 W/(m^2^ °C); (**d**) 500 W/(m^2^ °C); (**e**) 600 W/(m^2^ °C); (**f**) 700 W/(m^2^ °C).

**Figure 6:**
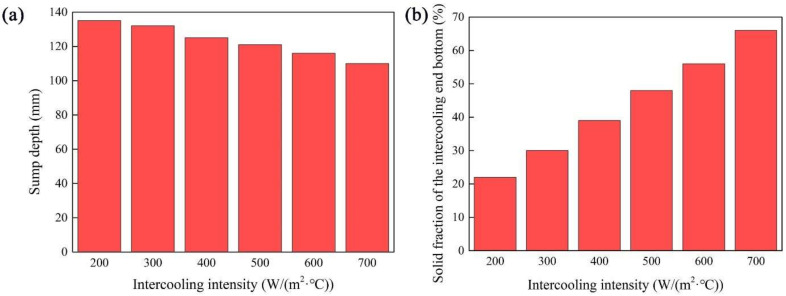
(**a**) Sump depth and (**b**) solid fraction of the intercooling end bottom.

The sump in the longitudinal section of the IEMS DCC billet is shown in Figure 5. With the increase in the intercooling heat transfer coefficient, the sump depth becomes shallower and the morphology becomes much gentler. The sump depth and solid fraction of the intercooling end bottom are measured, respectively, and shown in Figure 6. When the heat transfer coefficient increases from 200 W/(m^2^ °C) to 700 W/(m^2^ °C), the sump depth decreases from 135 mm to 110 mm, and the solid fraction of the intercooling end bottom increases from 22% to 66%. When the solid fraction of the intercooling end bottom reaches 50%, the risk of the internal stirrer solidifying into the billet increases. Hence, it is considered that the intercooling heat transfer coefficient should not exceed 500 W/(m^2^ °C), accordingly.

### 3.3. Microstructure

**Figure 7:**
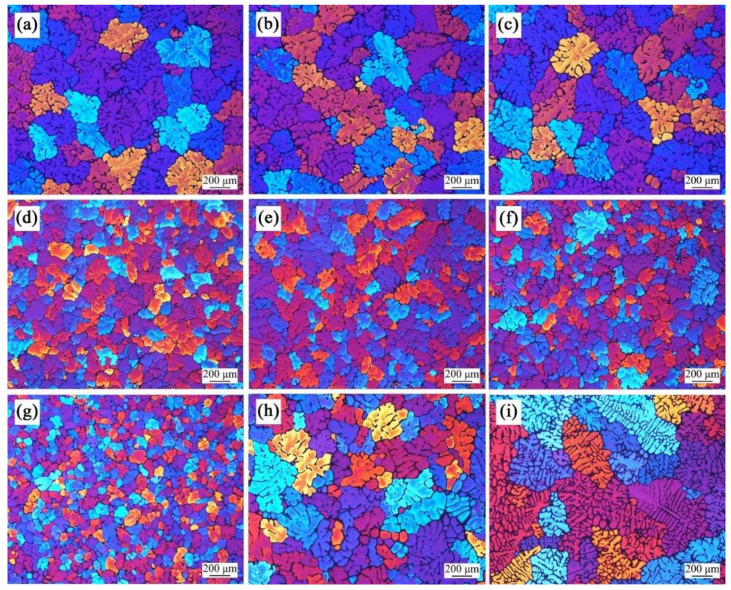
Microstructure in the edge (**a**,**d**,**g**), 1/2 radius (**b**,**e**,**h**), and center (**c**,**f**,**i**) of the billet under a liquid nitrogen flow rate of 80 L/min (**a**–**c**), 160 L/min (**d**–**f**), and 240 L/min (**g**–**i**).

Figure 7 shows the microstructure in the edge (a,d,g), 1/2 radius (b,e,h), and center (c,f,i) of the billet under a liquid nitrogen flow rate of 80 L/min (a–c), 160 L/min (d–f), and 240 L/min (g–i), respectively. When the liquid nitrogen flow rate is 80 L/min, the microstructure is very coarse; the average grain sizes are 297 ± 47 μm, 286 ± 41 μm, and 317 ± 56 μm in the edge, 1/2 radius, and center, respectively. The microstructure is refined significantly as the liquid nitrogen flow rate increases to 160 L/min. From edge to center, the average grain sizes decrease to 151 ± 13 μm, 159 ± 14 μm, and 149 ± 16 μm, respectively. It should be noted that the homogeneity of the microstructure has also been improved significantly. When the liquid nitrogen flow rate reaches 240 L/min, the microstructure in the edge maintains the refinement trend, with its average grain size decreasing to 108 ± 11 μm. Approaching the center, the microstructure becomes coarse and inhomogeneous, with the average grain sizes of 303 ± 71 μm and 399 ± 77 μm in the 1/2 radius and center, respectively.

**Figure 8:**
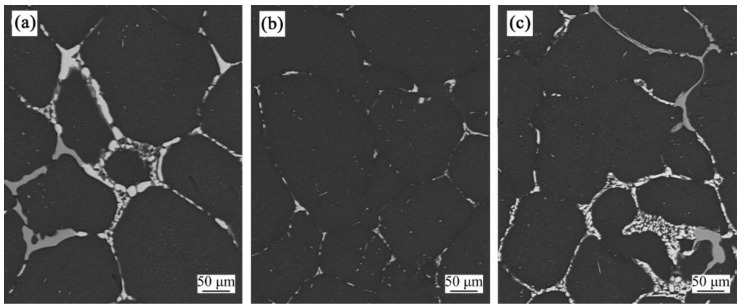
Second phases of the billet under a liquid nitrogen flow rate of 80 L/min (**a**), 160 L/min (**b**), and 240 L/min (**c**).

The 2219 Al alloy billets prepared by different liquid nitrogen flow rates were homogenized at 510 °C for 18 h, and their second phases are shown in Figure 8. A large number of eutectic compounds exist in the grain boundaries under a liquid nitrogen flow rate of 80 L/min and 240 L/min. These second phases are coarse, continuous, and netted, which cannot be completely eliminated by the heat treatment. When the liquid nitrogen flow rate is 160 L/min, more second phases are diffused into the α-Al matrix and fewer second phases remain in the grain boundaries.

### 3.4. Mechanical Properties

The 2219 Al alloys were solution treated at 535 °C for 8 h, quenched, and aging treated at 170 °C for 15 h, successively. The mechanical properties were tested and are displayed in Table 1. It is obvious that the mechanical properties increase as the grain size decreases, which follows the Hall–Petch formula. Tensile strength, yield strength, and elongation are enhanced from 329–338 MPa, 237–246 MPa, and 4–5% (liquid nitrogen flow rate of 80 L/min) to 373–379 MPa, 275–278 MPa, and 6–7% (liquid nitrogen flow rate of 160 L/min). Because the homogeneity of the microstructure sharply decreases under a liquid nitrogen flow rate of 240 L/min, the ranges of tensile strength, yield strength, and elongation are enlarged to 287–394 MPa, 203–302 MPa, and 3–8%, respectively.

## 4. Discussion

The refinement of IEMS can be attributed to the coupled function of EMS and intercooling. The EMS is generated as a result of interactions between magnetic fields and alternating currents. Its effects upon microstructure formation mainly include two aspects. Firstly, the free dendrites collide mutually under the Lorentz force, and the secondary dendrite arms are broken to form new nuclei [33,34]. Hence, the number of nuclei increases. Secondly, the flow of melt along the circle of mold is promoted by the EMS, and the heat transfer among the whole melt is accelerated [3,22]. Hence, the temperature gradient between the edge and the center decreases. Meanwhile, more heat can be removed via intercooling and a graphite ring, which contributes to relatively lower levels of temperature field [21]. As a consequence, the grains’ growth orientation is restrained, so more effective nuclei remain. Furthermore, the coarse columnar grains decrease and fine equiaxed grains increase.

When the intercooling liquid nitrogen flow rate is 80 L/min, the cooling intensity is small. Only the heat under and around the intercooling end can be removed. Therefore, the overall temperature of the melt is still high. The undercooling area is small and a few nuclei can be generated. However, some of them may flow into the superheated zone, resulting in a decrease in surviving nuclei [35,36]. As a result, the finally obtained microstructure is relatively coarse.

With the liquid nitrogen flow rates increasing to 160 L/min, the cooling effects enhance. The undercooling zone increases and more nuclei are generated. Meanwhile, the temperature field is relatively lower and more homogeneous, so more nuclei survive. Under the Lorentz force of IMES, the nuclei distribute more homogeneously in the sump. Moreover, the flow time of floating grains from the slurry zone to the sump bottom decreases because of the decrease in sump depth [37]. The growing time of those grains is restricted, which also promotes the refinement and inhomogeneity of microstructures.

When the intercooling liquid nitrogen flow rate reaches 240 L/min, some different characteristics appear in the microstructure. Firstly, the cooling intensity is further enhanced, meaning that more nuclei can be generated and transported with the melt flow into the edge of the billet. Therefore, it can be seen that the microstructure of the edge is much smaller than that of 160 L/min. Secondly, a large number of coarse dendrites exist in the 1/2 radius and center of the billet, with an average grain size much larger and more inhomogeneous than that of the edge. This can be explained as follows: the slurry becomes wider from the edge to the center, which increases the settling time of floating grains [38]. Meanwhile, the temperature below and around the intercooling end decreases as the liquid nitrogen flow rate increases. Hence, the viscosity increases accordingly. The floating grains flow, settle, and completely solidify in the sump bottom, which allows enough time for the floating grains to grow [39]. Hence, coarse microstructures have formed, some of which were even larger than 1 mm.

In the IMES DCC process, the t_1_, t_2_, t_3_, and t_4_ were measured and shown in Table 2. According to the formula (17), the heat transfer coefficients under a liquid nitrogen flow rate of 80 L/min, 160 L/min, and 240 L/min are 186 W/(m^2^ °C), 290 W/(m^2^ °C), and 387 W/(m^2^ °C), respectively. Meanwhile, the melt temperature where contacts with the intercooling end bottom were simulated and shown as follows: 624 °C for a heat transfer coefficient of 200 W/(m^2^ °C), 615 °C for a heat transfer coefficient of 300 W/(m^2^ °C), and 603 °C for a heat transfer coefficient of 400 W/(m^2^ °C). These temperatures can be considered as t_3_, approximately. Table 3 shows the t_3_ and heat transfer coefficient of experimental and simulated results. It can be inferred from the comparison that the experimental liquid nitrogen flow rate of 80 L/min, 160 L/min, and 240 L/min approximately correspond to the simulated heat transfer coefficient of 200 W/(m^2^ °C), 300 W/(m^2^ °C), and 400 W/(m^2^ °C), respectively.

## 5. Conclusions

The intercooling intensity is a primary parameter of IEMS DCC. Its effects on the temperature field and microstructure of large-scale 2219 Al alloy billets have been investigated by simulation and experimentation, respectively. Furthermore, the formation of microstructures under different intercooling intensities has been discussed. The main conclusions are as follows:

(1) The intercooling intensity significantly affects the temperature distribution and sump depth. The affected area increases and the sump depth decreases as the intercooling heat transfer coefficient increases. The heat transfer coefficient should not exceed 500 W/(m^2^ °C) because the solid fraction of the intercooling end bottom may exceed 50%.
(2) The average grain sizes in the edge, 1/2 radius and center are 151 ± 13 μm, 159 ± 14 μm, and 149 ± 16 μm, respectively, under a liquid nitrogen flow rate of 160 L/min, which are much finer than that of 80 L/min and more homogeneous than that of 240 L/min.
(3) The tensile strength, yield strength, and elongation under a liquid nitrogen flow rate of 160 L/min are 373–379 MPa, 275–278 MPa, and 6–7%, which is higher than that of 80 L/min and more homogeneous than that of 240 L/min.
(4) The relationship between experimental liquid nitrogen flow rates and simulated heat transfer coefficients has been determined. A liquid nitrogen flow rate of 80 L/min, 160 L/min, and 240 L/min approximately correspond to the heat transfer coefficients of 200 W/(m^2^ °C), 300 W/(m^2^ °C), and 400 W/(m^2^ °C), respectively.

## Figures and Tables

**Table 1:** Mechanical properties in the different positions of the billet under different liquid nitrogen flow rates.

Liquid Nitrogen Flow Rate	80 L/min			160 L/min			240 L/min		
Position	Edge	1/2 Radius	Center	Edge	1/2 Radius	Center	Edge	1/2 Radius	Center
Tensile strength (MPa)	335	338	329	377	373	379	394	336	287
Yield strength (MPa)	246	244	237	275	276	278	302	241	203
Elongation (%)	4	5	4	7	6	6	8	5	3

**Table 2:** Experimental t_1__,_ t_2,_ t_3,_ and t_4_ under different liquid nitrogen flow rates.

Liquid Nitrogen Flow Rate (L/min)	t_1_ (°C)	t_2_ (°C)	t_3_ (°C)	t_4_ (°C)
80	208	−105	617	442
160	173	−107	604	403
240	154	−108	595	384

**Table 3:** Comparison of experimental and simulated results.

Experimental Results			Simulated Results	
Liquid Nitrogen Flow Rate (L/min)	t_3_ (°C)	Heat Transfer Coefficient (W/(m^2^ °C))	t_3_ (°C)	Heat Transfer Coefficient (W/(m^2^ °C))
80	617	186	624	200
160	604	290	615	300
240	595	387	603	400

## Data Availability

Data sharing is not applicable to this article.
